# The causal effect of adipose tissue on Hodgkin’s lymphoma: two-sample Mendelian randomization study and validation

**DOI:** 10.3389/fimmu.2024.1400756

**Published:** 2024-05-30

**Authors:** Lihua Wu, Fei Liao, Xiangli Guo, Nainong Li

**Affiliations:** ^1^ Fujian Institute of Hematology, Fujian Provincial Key Laboratory on Hematology, Fujian Medical University Union Hospital, Fuzhou, China; ^2^ Translational Medicine Center on Hematology, Fujian Medical University, Fuzhou, China; ^3^ Department of General Surgery, Fujian Medical University Union Hospital, Fuzhou, China

**Keywords:** Hodgkin’s lymphoma (HL), non-Hodgkin’s lymphoma (NHL), adipose, body mass index (BMI), Mendelian randomization (MR)

## Abstract

**Background:**

Extensive research has been conducted on the correlation between adipose tissue and the risk of malignant lymphoma. Despite numerous observational studies exploring this connection, uncertainty remains regarding a causal relationship between adipose tissue and malignant lymphoma.

**Methods:**

The increase or decrease in adipose tissue was represented by the height of BMI. The BMI and malignant lymphoma genome-wide association studies (GWAS) used a summary dataset from the OPEN GWAS website. Single-nucleotide polymorphisms (SNPs) that met the criteria of P <5e–8 and LD of r^2 ^= 0.001 in the BMI GWAS were chosen as genetic instrumental variants (IVs). Proxy SNPs with LD of r^2^ > 0.8 were identified, while palindromic and outlier SNPs were excluded. Mendelian randomization (MR) analysis used five methods, including inverse-variance weighted (IVW) model, weighted median (WM), MR-Egger, simple mode, and weighted mode. Sensitivity assessments included Cochran’s Q test, MR-Egger intercept test, and leave-one-out analysis. Participants randomly selected by the National Center for Health Statistics (NHANSE) and newly diagnosed HL patients at Fujian Medical University Union Hospital were used for external validation.

**Results:**

The results of the MR analysis strongly supported the causal link between BMI and Hodgkin’s lymphoma (HL). The research demonstrated that individuals with lower BMI face a significantly increased risk of developing HL, with a 91.65% higher risk (OR_IVW_ = 0.0835, 95% CI 0.0147 – 0.4733, P = 0.005). No signs of horizontal or directional pleiotropy were observed in the MR studies. The validation results aligned with the results from the MR analysis (OR = 0.871, 95% CI 0.826 – 0.918, P< 0.001). And there was no causal relationship between BMI and non-Hodgkin’s lymphoma (NHL).

**Conclusions:**

The MR analysis study demonstrated a direct correlation between lower BMI and HL. This suggested that a decrease in adipose tissue increases the risk of developing HL. Nevertheless, further research is essential to grasp the underlying mechanism of this causal association comprehensively.

## 1 Introduction

Malignant lymphoma is a form of cancer that impacts the lymphatic system and is categorized by the type of cells involved and their level of maturity. The primary forms of lymphoma are Hodgkin’s lymphoma (HL), deriving from B cells, and non-Hodgkin’s lymphoma (NHL), which can originate from both B cells and T cells (1). HL is a unique form of blood cancer characterized by the presence of malignant Reed-Sternberg cells in an inflammatory setting. This condition commonly impacts individuals in their twenties and thirties, presenting with enlarged lymph nodes above the diaphragm and systemic B symptoms (2). The etiology of malignant lymphoma remains elusive, with no specific risk factors or causative agents identified. While familial history, viral exposure, and immune suppression have been implicated in the development of HL and NHL, the precise mechanisms underlying the disease are still unclear (3).

Adipose tissue plays a crucial role in Cancers, inflammation, and immunity by secretion of cytokines, hormones, and chemokines. These substances can both promote and reduce inflammation in the body, showcasing the active role of adipose tissue in regulating immune responses and inflammatory processes (4, 5). Excessive body weight, as indicated by body mass index (BMI), signifies an abnormal accumulation of fat in the body (6). A low BMI indicates a state of wasting or undernutrition. Hence, it is considered a valuable indicator for detecting obesity or wasting. Numerous studies have shown a link between a high BMI and an elevated risk of developing HL and NHL. This evidence indicates that obesity could be a potential risk factor for malignant lymphoma (7, 8). However, two significant studies have found that individuals who are obese or overweight have a more positive prognosis (9, 10). Therefore, the impact of BMI on malignant lymphoma remains controversial. Observational studies examining the relationship between BMI and malignant lymphoma may be influenced by biases like reverse causation and residual confounding, which can complicate our understanding of this association (11).

To address biases such as reverse causation and residual confounding, we employed Mendelian randomization (MR) to investigate the association between BMI and lymphoma. MR is a method that utilizes genetic variations as instrumental variables (IVs) to investigate whether a correlation between a risk factor and an outcome is indicative of a causal relationship (12). A two-sample MR estimates causal effects where data on the exposure and outcome have been measured in different samples (13). Currently, no previous studies have employed the MR method to examine the potential causal connection between BMI and susceptibility to malignant lymphoma. This research aims to address this gap by carrying out a two-sample MR analysis to ascertain whether there exists a causal relationship between BMI and the onset of malignant lymphoma.

## 2 Materials and methods

### 2.1 Data sources for BMI and lymphoma

We searched the MR Base database (http://www.mrbase.org/) (14), which houses a large collection of summary statistic data from hundreds of genome‐wide association studies (GWASs). We used the publicly available summary statistics data sets of GWAS meta‐analyses for BMI in individuals of European descent (n = 77482; Genetic Investigation of Anthropometric Traits consortium (GIANT consortium); https://gwas.mrcieu.ac.uk/datasets/ebi-a-GCST90095041/) (15) as the exposure. The genetic association studies databases for HL can be accessed on the FinnGen website (https://www.finngen.fi/en) (16). The genetic data for non-Hodgkin’s lymphoma (NHL) encompassed various subtypes, such as diffuse large B-cell lymphoma (DLBCL), follicular lymphoma (FL), mature T/NK-cell lymphomas, and other unspecified NHL types. This information has been rephrased for conciseness. These GWAS datasets are openly accessible and downloadable from the OPEN GWAS website (https://gwas.mrcieu.ac.uk/) (17). **Table 1** presented the relevant information for the outcomes.

**Table 1:** Characteristics of outcome.

Disease	Variable	Outcome GWAS ID	Cases	Controls	Source
**Malignant lymphoma**	Hodgkin lymphoma (all cancers excluded)	finn-b-CD2_HODGKIN_LYMPHOMA_EXALLC	369	180756	FinnGen
	Diffuse large B-cell lymphoma (all cancers excluded)	finn-b-C3_DLBCL_EXALLC	209	174006	FinnGen
	Follicular lymphoma (all cancers excluded)	finn-b-CD2_FOLLICULAR_LYMPHOMA_EXALLC	522	180756	FinnGen
	Mature T/NK-cell lymphomas (all cancers excluded)	finn-b-CD2_TNK_LYMPHOMA_EXALLC	150	180756	FinnGen
	Other and unspecified types of non-Hodgkin lymphoma (all cancers excluded)	finn-b-CD2_NONHODGKIN_NAS_EXALLC	533	180756	FinnGen

GWAS, genome‐wide association studies.

### 2.2 Genetic instrumental variant selection for BMI

**Figure 1:**
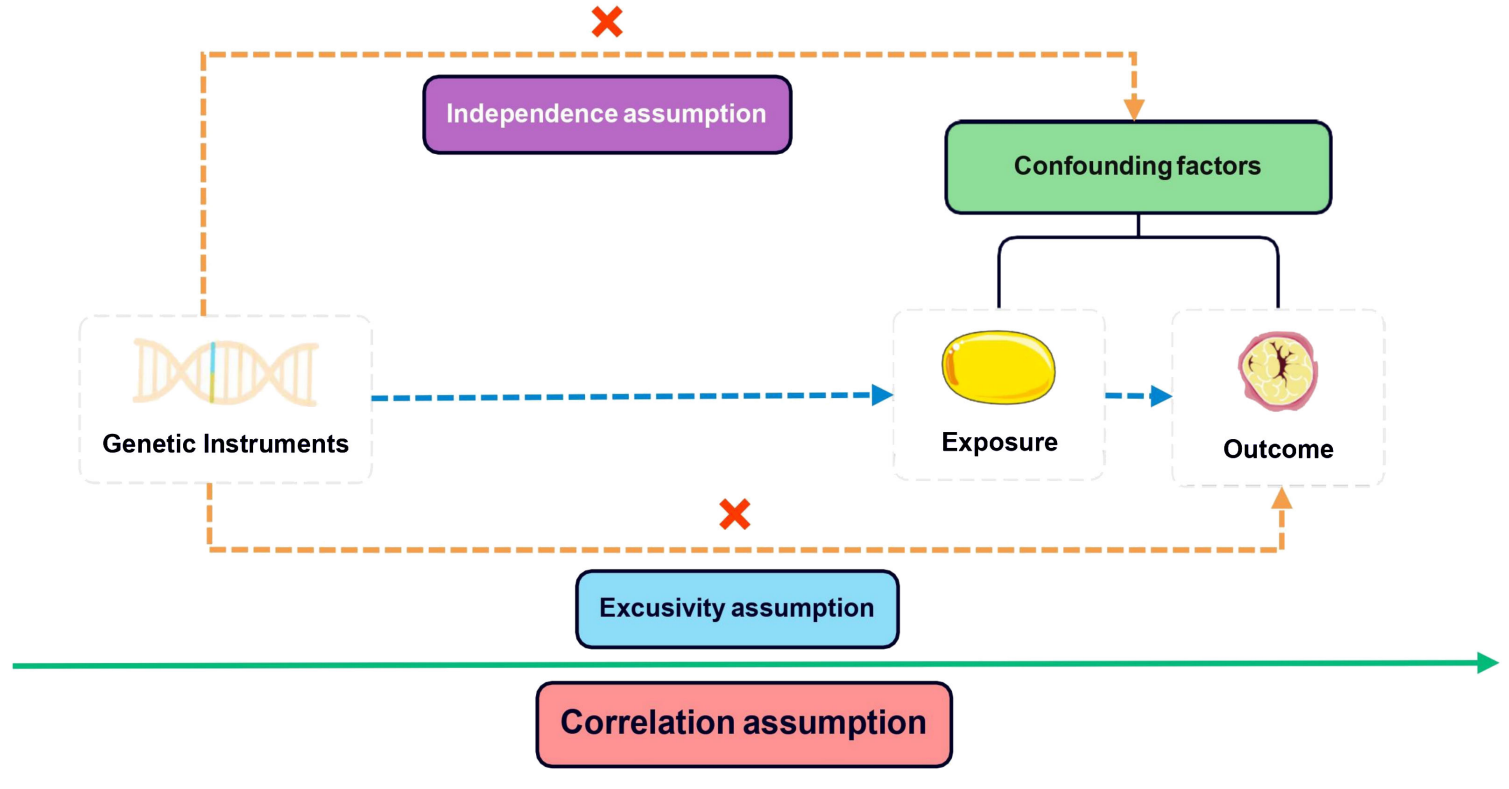
Three core assumptions of Mendelian randomization (MR) analysis.

In conducting MR studies, adherence to three fundamental assumptions is imperative: 1) a robust correlation between instrumental variants (IVs) and exposure variables exists; 2) the IVs are free from any influence of confounding factors about the exposure-outcome association; 3) genetic variants solely impact the outcome via the exposure route, excluding other pathways (18). The core assumptions depicted in **Figure 1** were addressed by following a systematic approach. Initially, IVs with genome-wide significance (P <5e–8) were extracted from the BMI GWAS. Subsequently, linkage disequilibrium (LD) was considered with a threshold of r^2 ^= 0.001 and a clumping distance of 10,000. Proxy single nucleotide polymorphisms (SNPs) with LD greater than 0.8 were then identified, while palindromic SNPs were excluded while harmonizing the BMI and HL GWAS datasets. Finally, the MR Pleiotropy RESidual Sum was employed to detect potential outlier SNPs and correct for any horizontal pleiotropy (19). The remaining SNPs were utilized for MR analysis in the subsequent stage. The F-statistic was calculated for each of the remaining SNPs to determine the strength of the genetic instruments. An F-statistic exceeding 10 signifies a robust genetic instrument essential for trustworthy MR analysis.

### 2.3 MR analysis

We employed five methods to tackle the issue of horizontal pleiotropy. These methods consisted of the inverse-variance weighted (IVW) model, weighted median (WM), MR-Egger, simple mode, and weighted mode. The primary analyses in this study utilized the multiplicative random-effects IVW method, known for providing accurate estimates when all SNPs are considered valid instruments. The weighted median method can also generate consistent estimates if more than 50% of the weight comes from valid instrument variants (20). The MR-Egger regression model was represented by the following formula:


$$\beta_{IV}=\alpha +\beta \cdot \left(1/\;SE\right)\;+\epsilon$$

**Figure 2:**
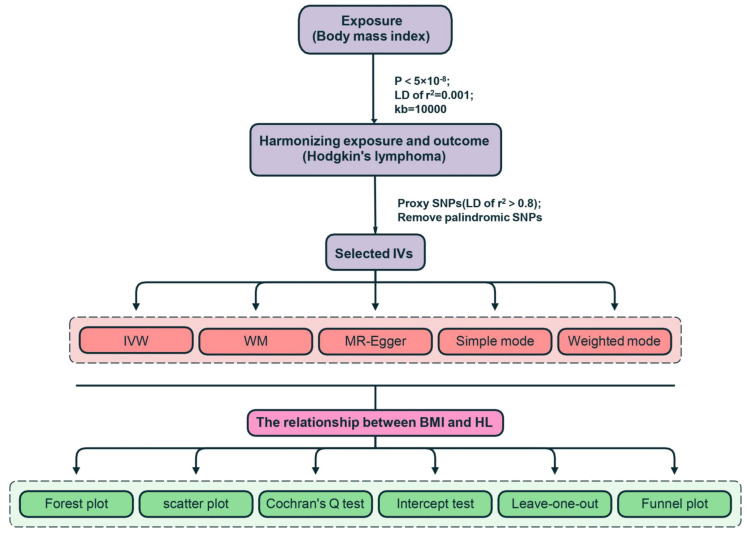
The flow diagram of the Mendelian randomization (MR) study.

In this equation, the components represented the following meanings: *β_IV_*: Effect size estimate for the instrumental variable (IV); *α*: Intercept term represented the average pleiotropic effect across all genetic variants; *β*: Slope term represented the causal effect estimate; *SE*: Standard error of the exposure; *ε*: Error term accounting for residual pleiotropy. MR-Egger regression offered estimates while accounting for horizontal pleiotropy, albeit with slightly reduced precision (21). MR-Egger was considered to be supportive when the effect estimate was consistent with MR-IVW. Through the utilization of these various techniques, we were successful in identifying and addressing the potential effects of horizontal pleiotropy in our study. The β Value acted as the effect size for determining the direction of causality, where β > 0 signifies the exposure as a risk factor for the outcome. Statistical significance was indicated by P< 0.05. The heterogeneity among genetic IVs was evaluated using Cochran’s Q test. Sensitivity analysis predominantly utilized the leave-one-out method. The causal link between BMI and lymphoma remained robust even when systematically removing one SNP at a time. The MR-Egger intercept test was employed to detect directional pleiotropy, with a P< 0.05 indicating its presence. Funnel plots were employed to assess directional pleiotropy, similar to their role in meta-analysis for detecting publication bias. The MR analysis was carried out using R software (version 4.3.2) with the package “TwoSampleMR” (version 0.5.10). The study’s flowchart is illustrated in **Figure 2**.

### 2.4 External verification

**Figure 3:**
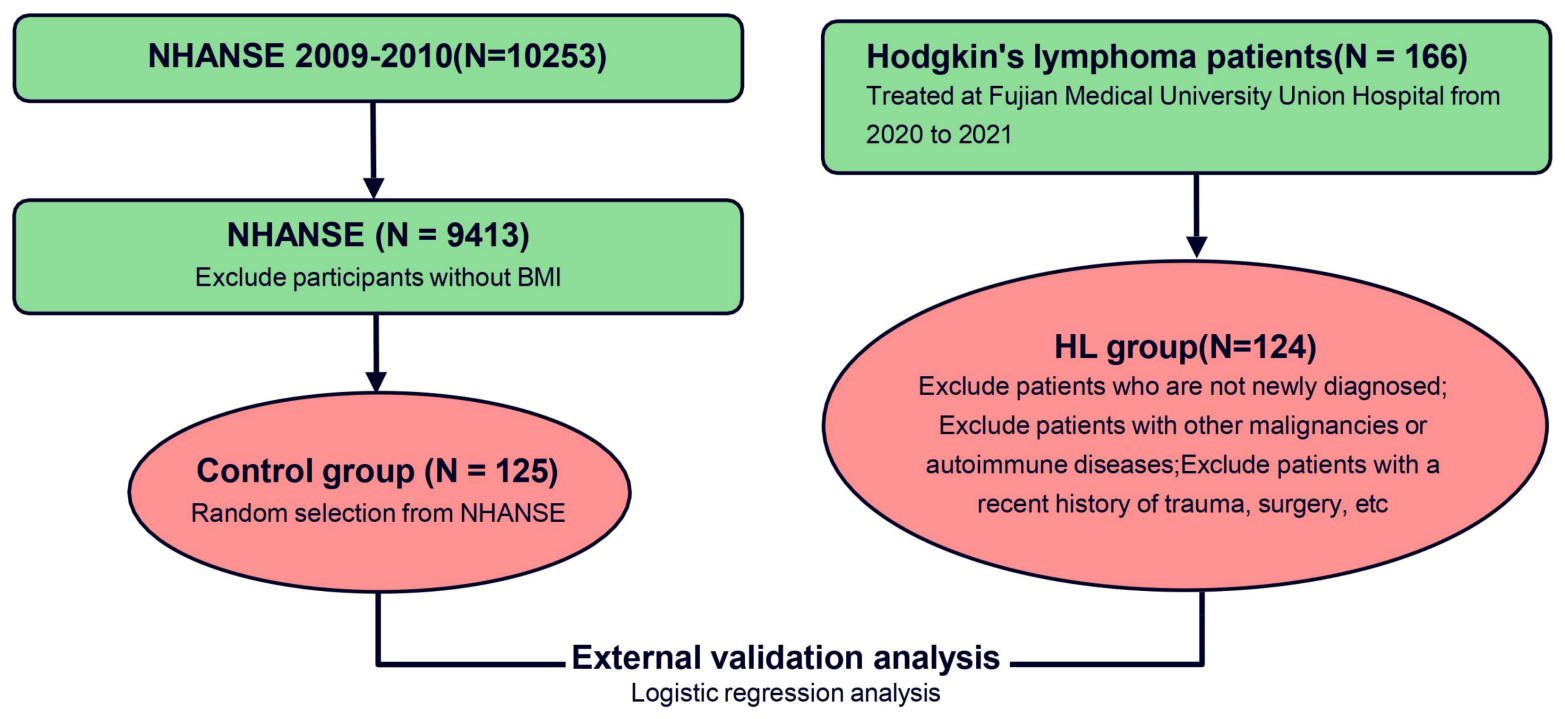
The flow diagram of the external verification.

A retrospective study was conducted at Fujian Medical University Union Hospital from 2020 to 2021, involving 124 newly diagnosed HL patients [Diagnostic criteria reference *Hodgkin lymphoma: 2023 update on diagnosis, risk-stratification, and management* (3)]. The validation study protocol was approved by the ethics committee of Fujian Medical University Union Hospital (Research project ethics approval number: 2021KJCX053). Our study excluded any personally identifiable information, and utilized BMI data gathered during routine clinical procedures. Minimal risks were associated with the study, safeguarding the subjects’ rights and interests and eliminating the necessity for individual informed consent. Control group data was sourced from the National Center for Health Statistics (NHANSE). NHANES, conducted by the National Center for Health Statistics of the Centers for Disease Control, is a comprehensive survey that gathers health and nutrition data from a nationally representative sample of individuals in the United States. The survey employs a rigorous stratified and multi-stage probability cluster design to ensure accurate and reliable results. All participants in the survey have given their informed consent to participate. In this study, we analyzed publicly available data from NHANES collected between 2009 and 2010. We randomly selected 125 people to be included in the external verification control group. Data analysis was performed using R software (version 4.3.2), and the correlation between BMI and HL was confirmed through a Logistic regression analysis. Statistical significance was established at P< 0.05. The study’s methodology is delineated in **Figure 3**.

## 3 Results

### 3.1 Instrumental variables for Mendelian randomization

We have selected 9 independent SNPs (rs10876528, rs1106529, rs1294409, rs1482852, rs224333, rs459193, rs7251505, rs7766106, rs979012) from GWASs on BMI as IVs (**Table 2**). For further details regarding these SNPs, researchers can refer to the National Center for Biotechnology Information (NCBI) Single Nucleotide Polymorphism Database (dbSNP: https://www.ncbi.nlm.nih.gov/snp/) (22).

**Table 2:** The baseline characteristics of the selected SNPs in the BMI GWAS.

SNP	Position	Gene	Chr	β	SE	P-value	EA	OA	EAF	F-statistics
rs10876528	54421476	*HOXC4/5/6*	12	0.036	0.005	6.91E-13	A	C	0.316	50.939
rs1106529	119531497	*TBX15*	1	0.037	0.005	5.36E-12	A	G	0.722	47.948
rs1294409	6738122	*LOC101928004*	6	0.025	0.005	3.25E-08	T	C	0.600	30.863
rs1482852	157080505	*LINC02029*	3	-0.028	0.005	2.35E-09	G	A	0.416	35.744
rs224333	34023962	*GDF5*	20	-0.037	0.005	1.88E-14	A	G	0.350	58.139
rs459193	56510924	*C5orf67*	5	-0.030	0.005	1.59E-09	G	A	0.766	35.764
rs7766106	127133993	*RSPO3/LOC105377989*	6	0.028	0.004	8.35E-11	T	C	0.508	41.660
rs7251505	33802542	*-*	19	-0.045	0.008	3.88E-08	A	G	0.102	30.453
rs979012	6623374	*-*	20	-0.026	0.005	4.69E-08	C	T	0.655	30.249

SNP, single nucleotide polymorphism; Chr, chromosome; β, beta coefficient for effect allele; SE, standard error for effect allele; EA, effect allele; OA, other allele; EAF, effect allele frequency.

### 3.2 The causal effect of BMI on HL

**Figure 4:**
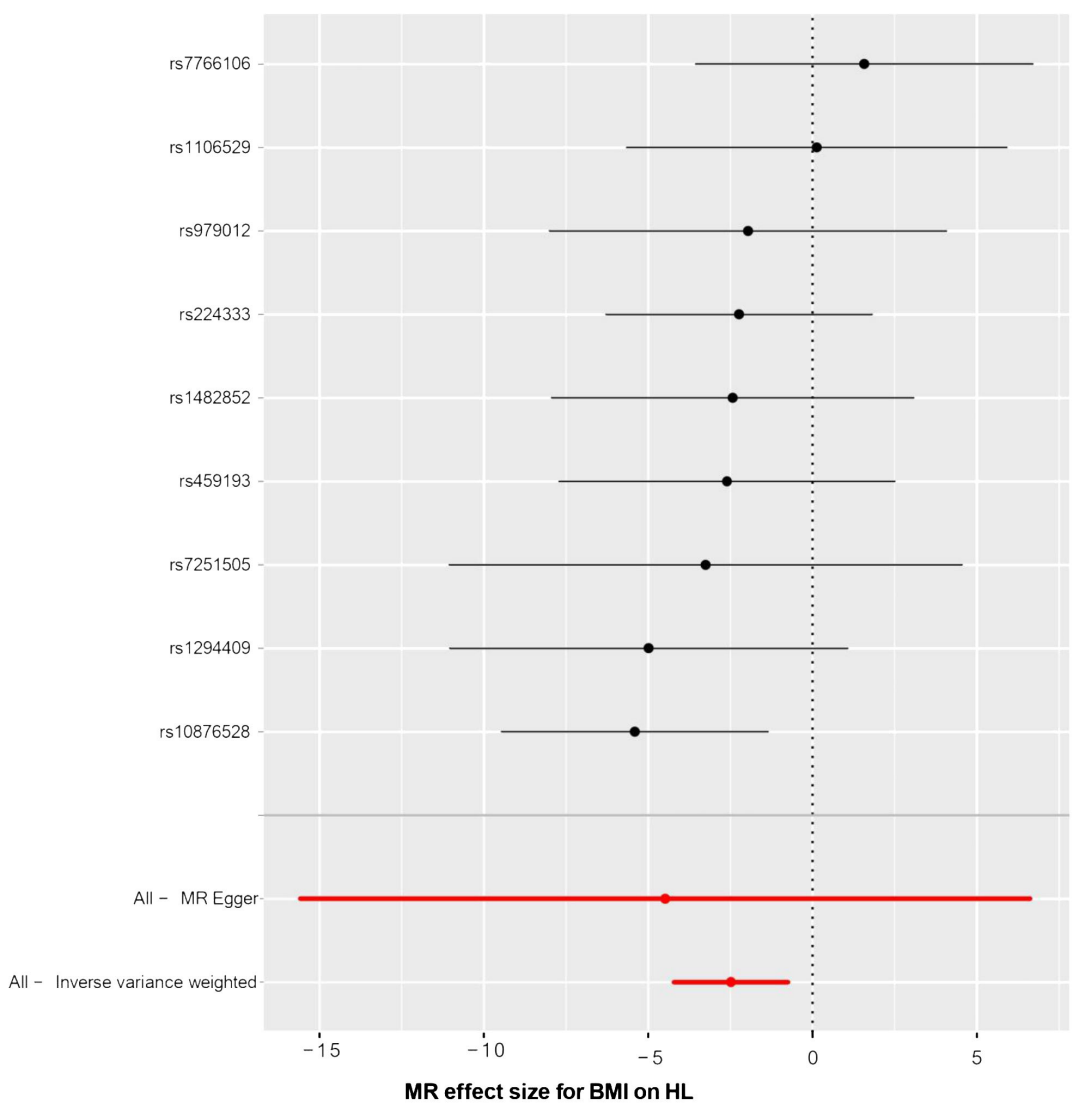
The forest plot of causality effect sizes of both single and merged single-nucleotide polymorphisms (SNPs) for BMI on HL. X-axis: β value.

**Figure 5:**
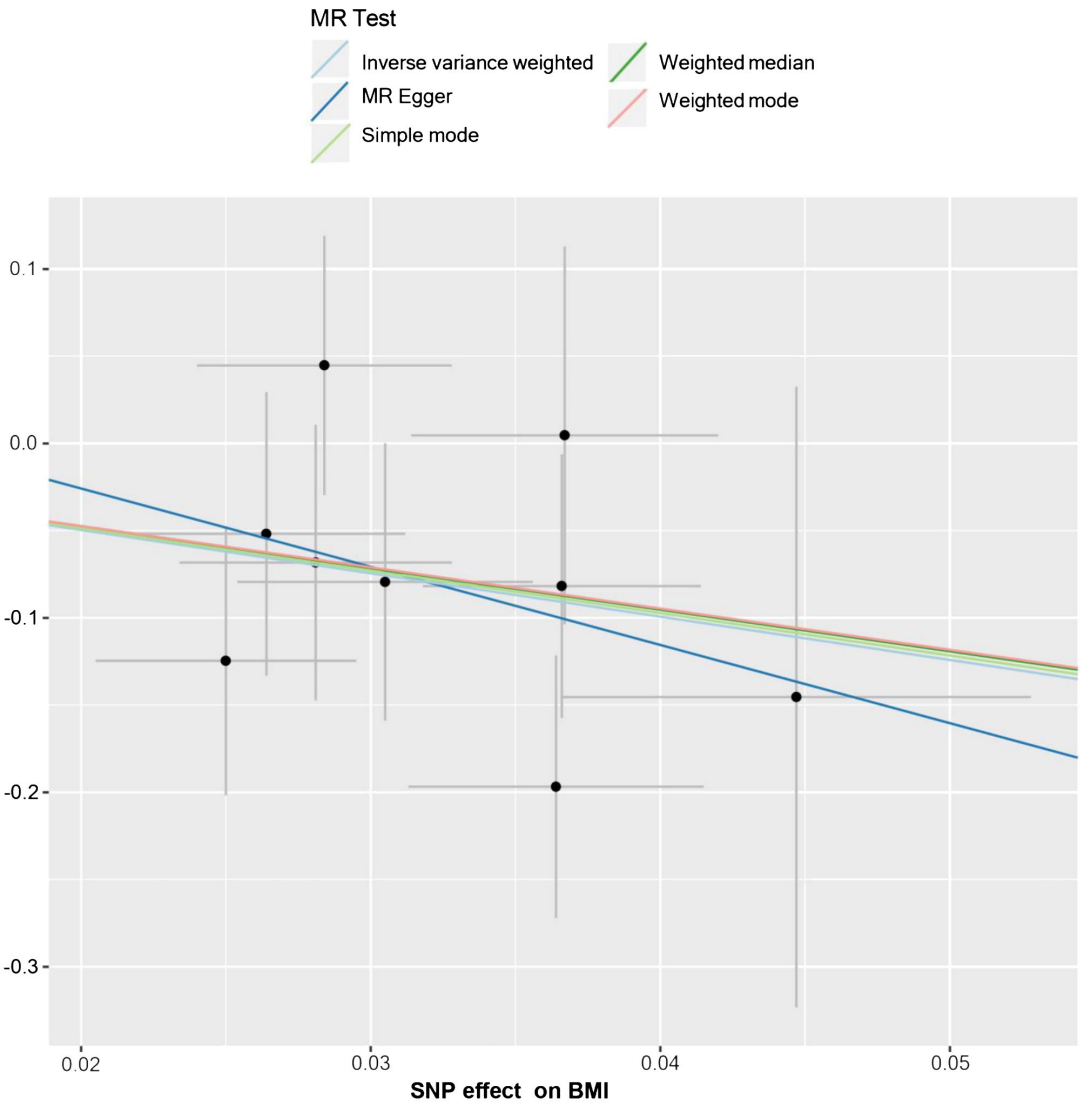
The scatter plot from genetically predicted BMI on HL. The X-axis represents the impact of SNPs on BMI, the Y-axis represents the impact of SNPs on HL. A negative slope less than 0 suggests that higher BMI is beneficial for the HL.

The relationship between BMI and HL has been confirmed through collective causal assessments (**Table 3**). Individuals with a higher BMI show a 91.65% reduced likelihood of developing HL (OR_IVW_ = 0.084, 95% CI 0.015 – 0.473, P = 0.005). Other Mendelian Randomization models also support this causal effect (OR_WM_ = 0.092, 95% CI 0.009 – 0.945, P = 0.045; OR_MR-Egger_ = 0.011, 95% CI 0.001 – 749.53, P = 0.455; OR_Simple-mode_ = 0.088, 95% CI 0.003 – 2.629, P = 0.198; OR_Weighted-mode_ = 0.094, 95% CI 0.002 – 3.656, P = 0.241). The consistency in the causal direction shown by these methods is remarkable, even though not all analysis methods yielded statistically significant results. However, with the P-values of IVW and WM both below 0.05, we maintain confidence in the MR analysis confirming the association between BMI and HL. Graphical representations like forest plots (**Figure 4**) and scatter plots (**Figure 5**) further illustrate the increased risk of HL in individuals with lower BMI. These findings were based on SNPs from the BMI GWAS and causal estimates from MR models.

**Table 3:** MR estimates from each method of assessing the causal effect of BMI on the risk of HL.

MR method	Number of SNPs	β	SE	OR (95%CI)	P-value	Q-d P-value
**IVW**	9	-2.483	0.885	0.084(0.015, 0.473)	0.005	0.656
**WM**	9	-2.383	1.187	0.092(0.009, 0.945)	0.045	
**MR Egger**	9	-4.482	5.664	0.011(0.001, 749.52)	0.455	0.564
**Simple mode**	9	-2.432	1.734	0.088(0.002, 2.629)	0.198	
**Weighted mode**	9	-2.367	1.869	0.094(0.002, 3.656)	0.241	

β, beta coefficient; MR, Mendelian randomization; SE, standard error; SNP, single nucleotide polymorphism; Q-d, Cochran’s Q-derived; IVW, inverse-variance weighted; WM, weighted median.

**Figure 6:**
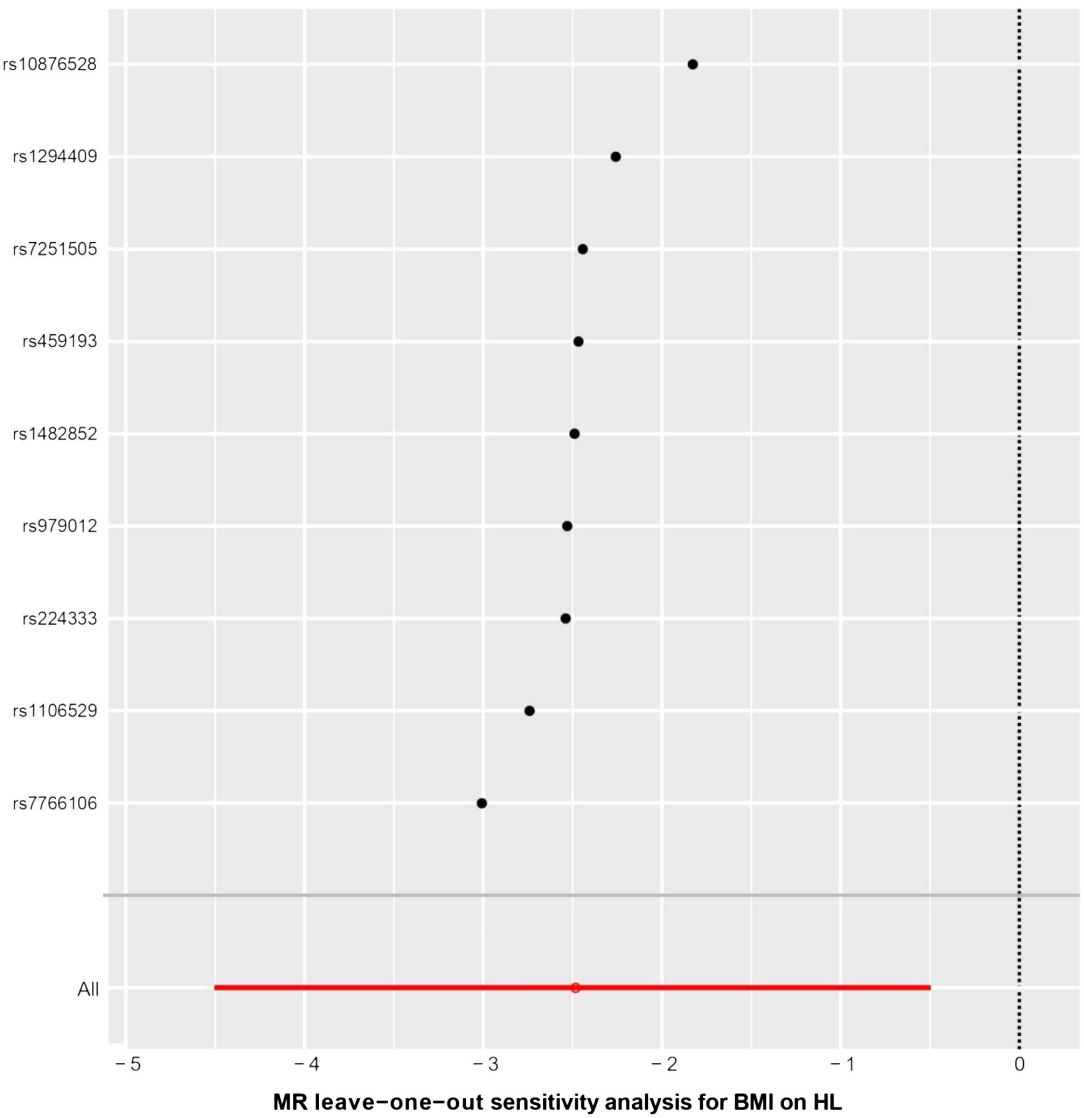
The results of leave-one-out methods for sensitivity analysis. X-axis: Estimated causal effects of the remaining SNPs after excluding one.

**Figure 7:**
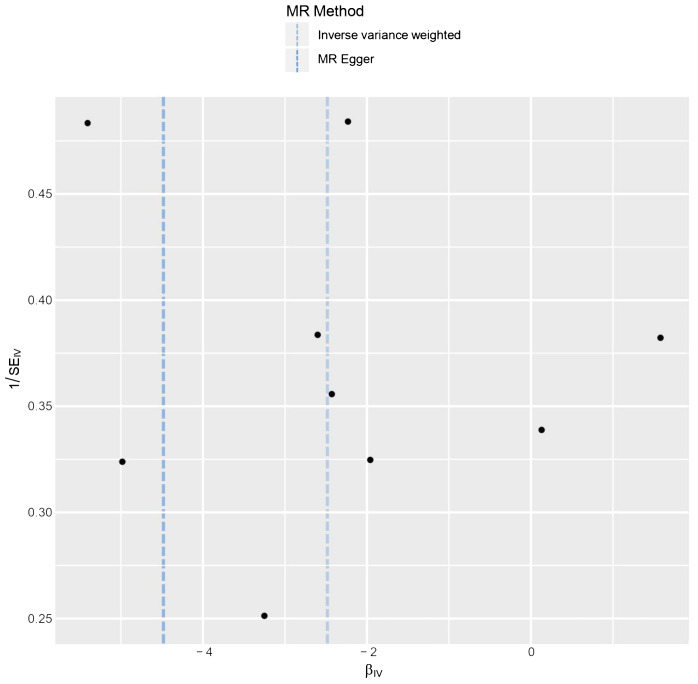
The funnel plot from genetically predicted BMI on HL.

The results of the statistical analysis showed no significant heterogeneity in the study on MR, with P-values of 0.564 for P_MR-Egger_ and 0.656 for P_IVW_. The MR-Egger intercept test indicated a p-value above 0.05, suggesting the absence of gene-directional pleiotropy. The sensitivity analysis, conducted through the leave-one-out method illustrated in **Figure 6**, consistently supported the conclusion on the causal relationship between BMI and HL, even when individual SNPs were excluded. Additionally, the symmetrical funnel plot in **Figure 7** indicated the absence of directional pleiotropy.

### 3.3 The causal effect of BMI on NHL

Malignant lymphomas are a complex group of cancers that can be classified into Hodgkin’s lymphoma (HL) and non-Hodgkin’s lymphoma (NHL) based on histopathological characteristics. NHL in particular exhibits a wide range of pathological patterns, immunophenotypes, clinical presentations, and responses to treatment (23). The outcome GWASs were classified lymphomas into specific subtypes of HL and NHL. These subtypes included diffuse large B-cell lymphoma (DLBCL), follicular lymphoma (FL), mature T/NK-cell lymphoma, and other unspecified types of NHL.

Two-sample MR analysis results indicated that there is no causal relationship between BMI and NHL (**Table 4**).

**Table 4:** The causal effect of BMI on non-Hodgkin’s lymphoma.

Exposure	Outcome	Results					
		MR methods	β	SE	OR	P-value	Q-d P-value
**BMI**	DLBCL	IVW	-1.561	1.168	0.210	0.181	0.583
		WM	-2.247	1.610	0.106	0.163	
		MR-egger	-8.000	7.471	0.000	0.320	0.561
		Simple mode	-2.903	2.524	0.055	0.283	
		Weighted mode	-3.101	2.497	0.045	0.249	
	FL	IVW	0.203	0.742	1.225	0.784	0.619
		WM	0.157	0.996	1.170	0.875	
		MR-egger	-4.647	4.747	0.010	0.360	0.637
		Simple mode	0.592	1.455	1.808	0.695	
		Weighted mode	0.283	1.326	1.327	0.836	
	Mature T/NK-cell lymphoma	IVW	1.993	1.380	7.338	0.149	0.590
		WM	1.474	1.846	4.367	0.425	
		MR-egger	1.640	8.816	5.155	0.858	0.481
		Simple mode	1.549	2.590	4.707	0.566	
		Weighted mode	1.363	2.630	3.908	0.618	
	Other and unspecified types of NHL	IVW	0.513	0.734	1.671	0.485	0.627
		WM	0.349	0.965	1.418	0.717	
		MR-egger	-4.110	4.696	0.016	0.411	0.637
		Simple mode	0.286	1.227	1.332	0.821	
		Weighted mode	0.239	1.237	1.269	0.852	

BMI, body mass index; WM, weighted Median; IVW, inverse variance weighted; SE, standard error; OR, odds ratio; Q-d, Cochran’s Q-derived; DLBCL, diffuse large B-cell lymphoma; FL, Follicular lymphoma.

### 3.4 External verification

**Figure 8:**
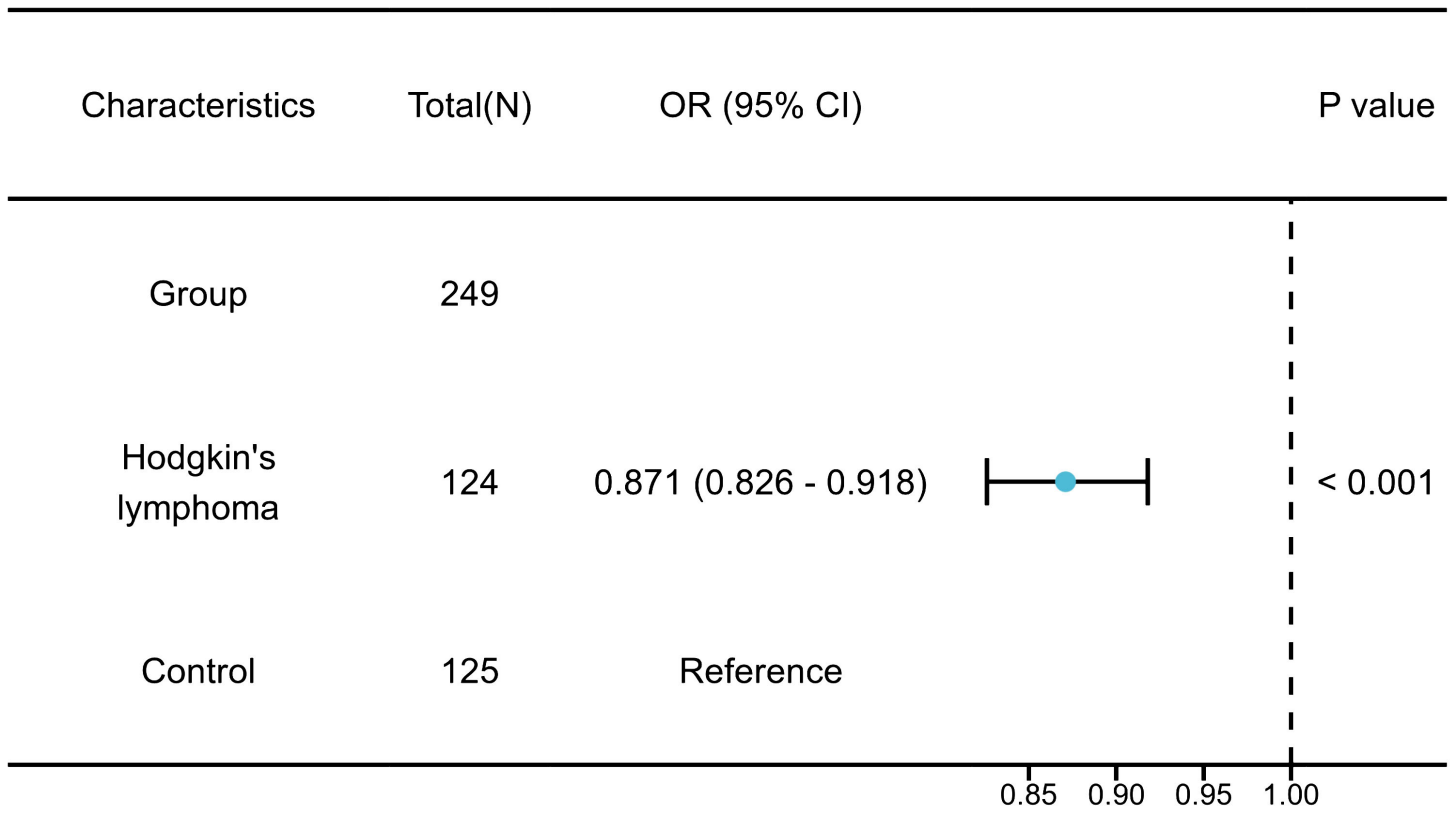
The forest plot of external validation.

External validation has confirmed a strong link between BMI and the onset of HL. Studies have indicated that individuals with a lower BMI face an increased risk of HL (OR = 0.871, 95% CI 0.8260 – 0.9180, P< 0.001) (**Figure 8**).

## 4 Discussion

This study is the first to examine the potential link between BMI and malignant lymphoma through a two-sample Mendelian randomization approach. In our study, we employed five different methods for estimating causal relationships in MR analyses: the inverse-variance weighted method, the weighted median method, the MR-Egger regression, the Simple mode, and the Weighted mode. We identified 9 SNPs significantly associated with BMI in the analysis. Our findings indicate a higher risk of HL in individuals with a lower BMI. Interestingly, no direct causal relationship was found between BMI and NHL in the study.

In today’s understanding of human metabolism, adipocytes are no longer seen as passive cells but as active endocrine and paracrine organs (24). By releasing adipokines, such as growth factors, cytokines, chemokines, and hormones, adipocytes have a significant impact on tissue angiogenesis and tumor formation (25). Including IL-6, IL-8, leptin, adiponectin, TNF-α, vascular endothelial growth factor (VEGF), osteopontin (OPN), haptoglobin (Hp), and YKL-40, among others (26–28). These molecules play a crucial role in influencing key mechanisms in cancer cells such as proliferation, apoptosis, and migration (29, 30). Therefore, BMI is associated with the development of many cancers, including breast cancer, colon cancer, and endometrial cancer (31). However, the role of high BMI in the development of HL remains controversial.

HL is characterized by an inflammatory microenvironment at the tumor site in lymph nodes (32, 33). The secretion of cytokines and chemokines by both HRS cells and surrounding non-neoplastic cells is believed to play a role in the growth and progression of HL (34). Additionally, adipocytes within the bone marrow microenvironment are thought to influence the behavior of HRS cells (35, 36). These adipocytes are a significant source of these adipokines, including leptin and IL-6 (37, 38), which activate the JAK/STAT pathway that is crucial for HL malignant cells (39, 40). The impact of adipocytes on the survival and proliferation of HRS cells through the secretion of adipokines is an area that requires further investigation, including whether adipocytes behave differently in the presence of HL malignant cells. Our research has shown that individuals with a higher BMI may have a lower risk of developing HL. We have identified 9 specific SNPs that are linked to this finding. The genes in which these SNPs are located may affect the development of HL by affecting the secretion of adipocytes, which helps explain why some obese HL patients have better prognoses. Further research into these genes could provide valuable insights into the connection between adipocytes and HL.

The study provides evidence of a causal relationship between BML and HL, but there are limitations to be aware of. The study primarily concentrated on a European population, which may hinder the generalizability of the findings to other regions, although using populations in the Americas and Asia for external validation. Furthermore, the reliance on data from GWAS meta-analyses restricted the ability to perform stratified analyses based on various demographics.

While our study does have some limitations, it also has several advantages. The wealth of data at our disposal allowed for a comprehensive analysis of incident HL and facilitated a robust genome-wide association study GWAS to pinpoint genetic instruments for MR analyses. In addition, various challenges may hinder the accuracy of analyses in MR studies, such as the potential for multiple downstream effects from a single genetic variant and non-random associations between genetic variants. To overcome these challenges, our study utilized strict criteria for IV selection and evaluated causality using five MR methods (IVW, WM, MR-Egger, simple mode, and weighted mode). Additionally, we conducted leave-one-out analyses to assess result robustness by excluding individual variants, and employed various sensitivity analysis to address pleiotropic effects and non-random associations of genetic variants. As a result, the causal relationships identified in our study can be considered reliable and robust.

## 5 Conclusion

According to the results of this MR study, it is suggested that lower BMI could be a notable risk factor for HL. This conclusion seems to suggest that our adipose tissue protects us from HL. Additional research is required to completely comprehend the underlying mechanism of this cause-and-effect relationship. This study acts as a prompt for internists to be cautious of the potential link between BMI and malignant lymphoma during patient care.

## Data availability statement

The original contributions presented in the study are included in the article/supplementary material. Further inquiries can be directed to the corresponding author.

## Ethics statement

This study used publicly available data from studies on human experimentation that have been approved by their respective institutional review boards. The validation study protocol was approved by the ethics committee of Fujian Medical University Union Hospital (Research project ethics approval number: 2021KJCX053). HL Group of validation excluded any personally identifiable information, and utilized BMI data gathered during routine clinical procedures. Minimal risks were associated with the study, safeguarding the subjects’ rights and interests and eliminating the necessity for individual informed consent.

## Author contributions

LW: Conceptualization, Data curation, Formal analysis, Investigation, Methodology, Software, Validation, Visualization, Writing – original draft, Writing – review & editing. FL: Conceptualization, Formal analysis, Investigation, Methodology, Software, Writing – original draft, Writing – review & editing. XG: Data curation, Investigation, Validation, Visualization, Writing – original draft, Writing – review & editing. NL: Conceptualization, Funding acquisition, Methodology, Project administration, Resources, Supervision, Writing – original draft, Writing – review & editing.
